# Can Different Admissions to Medical School Predict Performance of Non-Technical Skill Performance in Simulated Clinical Settings?

**DOI:** 10.3390/healthcare11010046

**Published:** 2022-12-23

**Authors:** Parisa Moll-Khosrawi, Wolfgang Hampe, Leonie Schulte-Uentrop, Christian Zöllner, Stefan Zimmermann, Thorben Huelmann

**Affiliations:** 1 Department of Anaesthesiology, University Medical Center Hamburg-Eppendorf, Martinistr. 52, 20246 Hamburg, Germany; 2 Institute of Biochemistry and Molecular Cell Biology, University Medical Center Hamburg-Eppendorf, Martinistr. 52, 20246 Hamburg, Germany

**Keywords:** emergency medicine, medical admission test, medical student selection, non-technical skills, patient safety

## Abstract

Non-technical skills (NTS) in medical care are essential to ensure patient safety. Focussing on applicants’ NTS during medical school admission could be a promising approach to ensure that future physicians master NTS at a high level. Next to pre-university educational attainment, many selection tests have been developed worldwide to facilitate and standardise the selection process of medical students. The predictive validity of these tests regarding NTS performance in clinical settings has not been investigated (yet). Therefore, we explored the predictive validities and prognosis of the Hamburg MMI (HAM-Int), HAM-Nat, PEA, and waiting as well as other quota (as example) designated by the Federal Armed Forces) for NTS performance in clinical emergency medicine training of medical students. During 2017 and 2020, *N* = 729 second, third, and fourth year students were enrolled within the study. The mean age of participants was 26.68 years (SD 3.96) and 49% were female students. NTS of these students were assessed during simulation scenarios of emergency training with a validated rating tool. Students admitted via waiting quota and designated by the Armed Forces performed significantly better than students admitted by excellent PEA (*p* = 0.026). Non-EU students performed significantly inferior (*p* = 0.003). Our findings provide further insight to explain how and if admission to medical school could predict NTS performance of further physicians.

## 1. Introduction

Traditionally, pre-university educational attainment (PEA) was used to select medical students. PEA has a high prognostic validity regarding academic success in medical school [1,2,3,4,5]. However, to facilitate and standardise the selection process of medical students, many selection tests have been developed worldwide [6]. These tests are expected to provide an incremental gain of knowledge in addition to the PEA. [7] The majority of these tests focus mainly on cognitive abilities, considering endpoints like examination results, drop-out rate, and duration of studies [1,4,8,9]. The “Hamburg Natural Science Test” (HAM-Nat) [10], “Test für Medizinische Studiengänge” (TMS) [3], “United Kingdom Clinical Aptitude Test” (UKCAT) [11], “BioMedical Admission Test (BMAT)” [12], or the “Medical College Admission Test” (MCAT) [13], are the most common tests in Europe which target cognitive skills. Highest incremental validities and correlations with study success have been shown for MCAT and HAM-Nat [14,15].

Next to cognitive tests, classical interviews [6], Multiple Mini Interviews (MMIs) [16] and Situational Judgement Tests (SJTs) [17] have gained a widespread popularity in medical school admission [18]. Although these tests are detached from medical or academic knowledge and mainly focus on psychosocial competencies, their predictive validity regarding non-cognitive abilities in clinical settings needs further investigation [18,19,20].

However, to ensure good patient care, it is important that the admission tests predict outcomes of interest, stretching beyond endpoints like semantic knowledge and recalling factual knowledge [21]. The performance of an individual in a clinical setting might be of greater significance than their results in a written test [21]. In accordance with this imperative, recent studies demonstrated the predictive validities for the BMAT and UKCAT regarding clinical practical and examination skills [21,22]. Further relevant outcomes need to be distinguished in future doctors are non-cognitive abilities in medical high-risk settings, such as emergency medicine [23,24,25]. The importance of these abilities has been emphasised repeatedly over the past years due to their essential impact on patient safety [26,27,28]. In the context of emergency medicine, these non-cognitive abilities are referred to as non-technical skills (NTS) [29]. NTS are defined as “the cognitive, social and personal resource skills that complement technical skills and contribute to safe and efficient task performance” [30]. They are detached from medical knowledge or technical skills and do not enhance with growing clinical experience [31]. Therefore, the early training of NTS already in undergraduate medical education has been demanded and supported [24,32,33].

Every physician is at risk of being confronted with life-threatening emergencies among their patients. Therefore, in order to prevent adverse events, NTS are essential for many medical specialties and not only limited to high-risk medical fields, such as anaesthesiology or emergency medicine [34].

Despite many educational efforts, the recent European Resuscitation Council Guidelines 2021 (ERC) identified a gap in knowledge and evidence regarding the best way to train and convey NTS [27]. NTS are acquired among the socialisation process of every human and are non-homogeneously dispersed [35]. Furthermore, NTS are skills which might be detached from being gained through deep learning or semantic efforts [36]. For high levels of NTS, knowledge has to be integrated and transferred into action and hereby, the expression of personality traits might be of even greater significance than educational strategies [36]. To ensure that future physicians master NTS at a high level, focusing on applicants’ NTS during medical school admission could be a promising approach [35]. Therefore, interest in the early identification of future doctors’’ NTS, already during admission to medical school, is increasingly growing [6,37]. So far, no published study has investigated the predictive validity of admission tests regarding NTS performance in a clinical setting [8].

To summarise, the early identification of students with NTS attributes is a promising approach, but knowledge about the predictive validity of admission tests that ensure these skills is scarce. Therefore, this prospective longitudinal study explored the predictive validities and prognosis of the Hamburg MMI (HAM-Int), HAM-Nat, PEA, and waiting quota for NTS performance in the clinical emergency medicine training of medical students in different years of medical school.

## 2. Methods

### 2.1. Study Design and Setting

We performed this prospective longitudinal study at University Medical Center Hamburg-Eppendorf from 2017 to 2020. Medical students who participated in the mandatory simulation-based emergency trainings of the Department of Anaesthesiology in their second, third and fourth years of medical school were sampled.

The undergraduate medical curriculum of the medical faculty of Hamburg is structured based on the spiral curriculum design described by Harden [38]. The learning contents of preceding teaching units are revisited and expanded in the subsequent teaching units. Aligned to this curriculum design, the anaesthesiology/emergency medicine teaching units take place from the first to the fourth year of medical school and build upon each other. Basic Life Support (lecture, seminar, mannequin-based training) is part of the first year, followed by Trauma training and Advanced Cardiac Life Support I (lecture, seminar, high fidelity simulation-based training) in the second year. Advanced Cardiac Life Support II (lecture, seminar, high fidelity simulation-based training) and operating room simulation (OR-SIM, adverse events in the operating theatre) (lecture, seminar, high fidelity simulation-based training) are part of the third year. In their fourth year of medical school, the students participate in Advanced Cardiac Life Support III (lecture, seminar, high fidelity simulation-based training).

A total of twelve–eighteen students are assigned to each high-fidelity simulation-training. Each training has a predefined set of standardised scenarios, through which the students rotate in randomly chosen small groups. The scenarios are standardised and solely for each type of training session. Each scenario is executed in a different room of the teaching facility. Three students of each small group form the emergency response team: one of the students partakes the role of the emergency physician/anaesthesiologist; two students partake the roles of paramedics/anaesthetic co-workers. Each scenario is supervised by an instructor (anaesthesiologist), who is experienced in medical teaching and emergency medicine. High-fidelity simulators (Resusci Anne, Laerdal, Scavenger, Norway) are used, which are suitable to create real-life settings and to train technical skills such as drug administration, endotracheal intubation, or defibrillation. The scenarios are composed of emergency situations, leading to cardiac arrest. With each subsequent simulation-based training the scenarios become more complex, requiring higher levels of technical and non-technical skills. To rule out the theory that NTS are influenced and biased by a lack of theoretical knowledge, each training session starts with a short 30 min seminar to refresh the theoretical knowledge of emergency medicine and cardiac life support among participants. Principles of Crew Resource Management (CRM) are addressed briefly; the role distribution and the corresponding tasks are clarified (physician, paramedic/anaesthetic co-workers).

At the beginning of each scenario, the emergency response team (small group of students) is provided with information on the emergency. This information is in accordance with a real-life setting and consists of hard facts, such as “cardiac arrest on the ward number 4”. The student in the role of the physician is supposed to lead the response team. This task includes to indicate and delegate necessary medical procedures and provide the medical care alongside with the team. Each scenario is followed by a debriefing which is conducted by the instructor, to provide emotional support and to talk through the medical facts. The debriefing is composed of three conceptual phases: gathering, analysing, and summarizing. The role of the instructor is that of a teacher, meaning that the debriefing is held in a conventional way.

### 2.2. Participants

Second, third and fourth year students who participated in the mandatory anaesthesiology teaching units of their medical school year were enrolled within the study (*n* = 729). Eligibility criteria were previous participation in “Basic Life Support” (1st year) and in “Trauma training” (2nd year), to rule out that unfamiliarity with the teaching format caused emotional stress and cognitive overload and hence caused bias in the study outcomes [39]. An overview of the demographic data of the study participants is provided in Table 1.

The study population was selected to medical school prior to 2020, before the changes of the German Federal Constitutional Court regarding admission to German medical schools were implemented. During the study period, medical schools had to select 40% of the applicants based on quotas such as waiting list, excellent PEA, students from non-EU nations, and other groups (hardship, medical officers from the Federal Armed Forces). The waiting list includes the applicants that have waited longest for admittance since high-school graduation. For the remaining 60% of the study places, the German medical schools were free to select the students by the procedure of their own choice. At Hamburg Medical School the first 100 places of this 60% quota were assigned to those applicants who had received the best PEA in combination with the best result of the HAM-Nat. The following about 100 places were assigned to applicants who also took part in the selection interviews (HAM-Int).

Demographic data of the study participants were collected independently from the study in a database after acceptance to medical school. Prior to each semester of the study period, the students were contacted with information on the study. Participation in the study was voluntary and written informed consent was obtained from each participant.

### 2.3. Outcomes

NTS were assessed using the “Anaesthesiology students’ non-technical skills” (AS-NTS) [40], which was developed to rate medical students’ NTS during simulation-based training. The AS-NTS has been broadly validated regarding feasibility, inter-rater reliability and has proven to be applicable independently from the rater’s experience in emergency medicine or medical education. Reliability of the AS-NTS has also been proven to be high (mean Cronbach’s alpha 0.88 for all subscales). Furthermore, it has been reported that the AS-NTS covers all the necessary NTS for student emergency education [40]. For each scenario of each training session, the instructor filled out the AS-NTS for the student partaking the physicians’ role. The AS-NTS is composed of three dimensions:

- Planning tasks, prioritising, and problem-solving;
- Teamwork and leadership;
- Team orientation.

Performance is rated on a five-point Likert scale (1 = very good; 5 = very poor). An underlying skill structure is used to give behaviourally anchored rating examples to clarify what a “good” or “poor” performance on each dimension might look like.

### 2.4. Statistical Analysis

For all data descriptive statistics (standard deviations, means) were calculated.

The data were analysed using linear regression models. For the admission variable average treatment effects were calculated with Excellent PEA as the reference group. Modelfits were established via a global F test and the inspection of the adjusted R^2^. Furthermore ANOVAS (analysis of variances with multiple testing) were carried out as global tests. All calculations were performed in R version 4.1.2. [41].

## 3. Results

### 3.1. Participants

A total of *n* = 729 students participated in the study. Data from *n* = 620 students were included in the final analysis, who at least took the role of the physician one time during the simulation scenarios (the AS-NTS was only filled out for the physicians of each simulation scenario). From these students, 104 were admitted via waiting quota, 83 via excellent PEA, 192 via HAM-Nat, and 133 via HAM-Int. Further 18 non-EU students, 22 students who were designated by the Armed Forces, and 68 through the “other” quota were included.

### 3.2. Outcomes

A total of *n* = 1172 AS-NTS assessments were conducted. *n* = 72 assessments had to be excluded due to missing data. Table 2 summarises the admission of participants and the number of included AS-NTS ratings for each admission group as well as their mean age.

The overall performance of the whole group of students is depicted in Table 2.

Overall, the students improved their NTS during the study period, beginning with their worst performance on all AS-NTS dimensions at the first assessment (ACLS I). The best performance of NTS was assessed during the third training (OR-SIM) which declined slightly at the last assessment (ACLS III). The analysis of variances (ANOVA) showed significant differences of NTS performance between the training (*p* < 0.01) and significant differences between the points of the three dimensions of the AS-NTS (*p* = 0.016).

**Figure 1:**
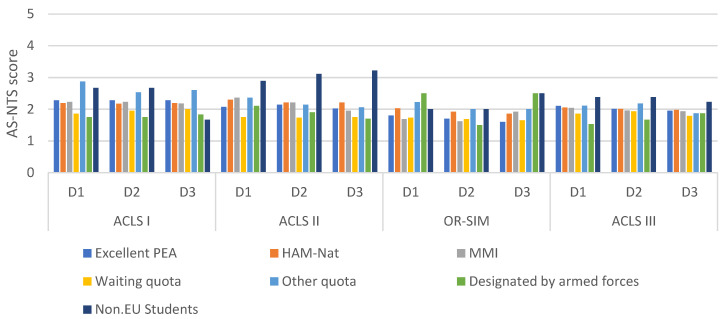
NTS performance of the investigated cohorts for each training session and each dimension of the AS-NTS. Abbreviations. MMIs = Multiple Mini Interviews; AS-NTS = Anaesthesiology students’ non-technical skills. ACLS = Advanced Cardiac Life Support. OR = Operating room. D = Dimension of AS-NTS. D1: Planning tasks, prioritising and problem-solving; D2: Teamwork and leadership; D3: Team orientation. Note: A low numeric AS-NTS score complies with a better NTS performance.

The comparison of NTS performance of the different cohorts of students indicated that students admitted via waiting quota and designated by the Armed Forces, performed significantly better than students admitted by excellent PEA (*p* = 0.026). Non-EU students showed significantly inferior performance on all AS-NTS dimensions (*p* = 0.003). The students admitted via HAM-Int performed slightly better on all the three NTS dimensions and the students admitted via HAM-Nat performed slightly inferior to the students admitted via excellent PEA. These differences were not statistically significant (*p* = 0.956 and *p* = 0.604) but relevant based on the global testing, which revealed more variances than there might be solely due to coincidence: Adjusted R² = 0.05, DF = 1087, F-Statistic = 7.055, *p* < 0.001 (Figure 1, Table 3).

To rule out the age of the participants as a confounding factor (on average, a.e. students from the waiting quota are older than students from the excellent PEA Quota), it was included in the statistical model but did not show a significant impact.

## 4. Discussion

In this prospective longitudinal study, the predictive validities of MMIs, HAM-Nat, PEA and waiting quota, regarding NTS performance in a simulated, real-life clinical setting were explored. We found that students admitted by waiting quota and designated by the Armed Federal Forces performed significantly better and non-EU students performed significantly inferior to the other students.

So far, only few admissions have been validated regarding professional success [21,22] and most investigations targeted endpoints which are linked to intellectual or cognitive abilities [1,4,21,42]. However, next to cognitive abilities, modelling studies have demonstrated that non-cognitive abilities, such as personality traits (conscientiousness) mediate academic performance [43,44]. Cognitive abilities might be even inferior predictors of clinical performance than the amount of relevant semantic knowledge [45]. In the context of emergency care, these non-cognitive skills are referred to as NTS. However, evidence on the predictive validity of medical school admission and performance of NTS in a simulated clinical emergency setting is scarce. It has been demonstrated that MMI results and supervisor ratings on communication skills during emergency medicine do correlate [46], but no published study targeted NTS as a whole construct. As NTS have been identified to be an important component ensuring and increasing patient safety [28], it would be preferable to target NTS of future doctors already in the selection process of medical schools [24]. Although the differentiation between non-cognitive and cognitive skills is challenging [47], MMIs are specifically designed to target non-cognitive skills [48]. In particular, the Hamburg MMI assesses psychosocial competencies, predominantly in simulation scenarios which are specially designed to target interpersonal skills (a.e. communication) [49]. Furthermore, since 2016, teamwork skills have been integrated into the dimensions of the MMI. Teamwork skills are composed of leadership and team orientation [50], which are important components of required NTS in emergency medicine. Therefore, they are targeted by many NTS taxonomies, as well as the AS-NTS [40,51]. Based on the design of the MMIs, it could be deduced that the MMI-selected students [8,52] would also display good NTS performance in the simulated clinical emergency settings. Contrary to our postulations, our results indicate no superior NTS performance of MMI-selected students. Therefore, the MMI seems not to measure competencies that are relevant in the practical context of emergency situations. These findings can be explained with several considerations. Based on interactionism, a social psychology theory, peoples’ behaviour in a situation is influenced by the environment, as well as on inter-individual interactions [53]. Therefore, the characteristics of situations determine how traits are expressed in behaviour. As the emotional stress of emergency simulation training is different than the stress during the MMI, the variability in trait-expressive behaviour within the two settings is plausible [54]. The same explanation can be transferred to the positive findings of Knorr et al., who—contrary to our study—did find positive predictive validities of MMIs and general practitioner evaluations on psychosocial competencies of students [48]. However, although NTS are the backbone of good patient care in critical situations, the question how to convey and train these skills remains unanswered [27]. It could be assumed, that NTS relevant for emergency medicine might underlie a far more complex structure of behavioural traits, than daily psychosocial competencies. Therefore, the MMI may assess a more subjective impression of general psychosocial competencies while the AS-NTS targets more objective competencies in a more complex scenario.

Excellent PEA, HAM-Nat, or HAM-Int scores require diligence and learning efforts and therefore, it could be reasonably assumed that these students have good NTS performance in emergency simulations. Nevertheless, we did not find superior NTS performance of neither MMI-nor PEA or HAM-Nat selected students. A possible explanation might be that academic success and performance of practical skills are positively influenced by diligence and learning effort but NTS are detached from these efforts [36]. In support of this argument, recent approaches from genetic research, that identified the genetic basis for non-cognitive skills by separating educational success from intellectual levels, merits consideration [55].

Other factors may influence the individual expression of non-cognitive skills and these personal skills are non-homogeneously distinctive as they are acquired among the socialisation progress of every individual [35]. The expression of NTS might depend on personal traits like conscientiousness [30], which is a strongly predictive personality factor for clinical performance, as well as NTS [9,56,57]. Conscientiousness increases with age [56]; therefore, we assumed that the students who were admitted via waiting quote, would perform better due to higher levels of conscientiousness and consecutively NTS. Although those students showed a superior performance, the statistical analysis of influencing factors showed that age itself is not determining the expression of NTS and the relationship between age and NTS is not linear. The determining factor for NTS expression showed to be the admission quota to medical school.

As stated before, it is important that admission tests predict relevant postgraduate outcomes of future doctors and ideally, they are assessed in postgraduate training during the actual clinical phase [21]. Therefore, one might question our study design. We targeted NTS in undergraduate training, while using simulation-based training as a proxy for a clinical setting. Linking admission results to actual clinical performance is defying. Nevertheless, our results are transferable to postgraduate performance to a certain extent. The argument against the study setting can be ruled out, when considering how standardly NTS are trained and assessed. In high-risk settings, such as aviation and nuclear power plants, NTS are key elements for licensing and are assessed in simulation-based training [58,59,60]. Much the same applies for emergency medicine. Even in specialty training, NTS are assessed and trained in simulation-based settings, because the real clinical setting has not proven to be adequate for this purpose [34]. Therefore, using a simulation-based setting is a plausible proxy to assess actual clinical behaviour. The question if high levels of NTS assessed in undergraduates, also predict the levels of NTS in their later professional life, remains unanswered. Therefore, the generalization of our results is not entirely possible. Nevertheless, as NTS do not enhance with clinical experience [31], it can be concluded that the personality traits and their expression already at student age might have a significance for later professional life. As an example, it seems unlikely that a student who is conscientious becomes a less conscientious doctor. This postulation is supported by findings from behavioural psychology, which state that people tend to become even more conscientious as they age [61]. Conscientiousness is directly linked to NTS [30]; hence, our results become more generalizable. Furthermore, even philosophical views of personality traits conclude that pieces of personality cannot be changed, only the change of the whole identity would affect the expression of personality traits. These identity changes would only take place under special circumstances and are not the normative pattern [61,62].

A further possible limitation of our study should be acknowledged. We restricted our analytic samples as they were preselected. At Hamburg Medical School, the first 100 places of the volatile 60% quota were assigned to those applicants who had received the best PEA in combination with the best result of the HAM-Nat. The following about 100 places were assigned to applicants who also took part in the selection interviews (HAM-Int). For example, applicants with best PEA in combination with best HAM-Nat results, did not participate in the HAM-Int. While our results are likely to be generalizable to the admission tests, we cannot rule out different results if every applicant would have had participated in all admission tests.

One might argue that the comparison of AS-NTS performance of medical students in different years of medical school is questionable. However, this argument can be mitigated, because we compared the cohorts of medical students in each year of their training. Furthermore, the assessment of NTS with the AS-NTS, as it is described by the authors, allows the comparison of different and repeated measurements at different time points of training [40]. Additionally, the scenarios of the emergency training are designed aligned to the spiral curriculum [38], meaning that with each proceeding training, the amount of necessary technical as well as non-technical skills are greater. Hereby, the different training or the NTS of different students in different years of medical training can be compared. Through this, one important strength of our study is derived: while exploring the predictive validities of different admissions regarding NTS performance, we systematically measured NTS with a validated tool, which has been developed specifically for undergraduate education.

## 5. Conclusions

Our findings open new perspectives regarding the complex structure and predictive validities of different admissions to medical school regarding NTS performance. However, it remains unexplained how and if admission to medical school can predict NTS performance in clinical emergency settings. Therefore, more research is needed to determine how admission to medical school can predict clinical NTS performance and whether admission tests need to be adapted accordingly.

## Figures and Tables

**Table 1:** Demographic data of study participants.

Admission	Number of Students Enrolled (*n* = 674)	Mean Age in Years	Standard Deviation of Mean Age	Percentage (%) of Female Students
All admissions	674	26.68	3.96	49
Excellent PEA	93	24.65	2.08	59
Ham-NAT	207	25.04	2.10	40
Ham-INT	153	25.10	2.53	69
Waiting quota	108	32.78	3.55	57
Other quota	72	28.10	3.23	53
Designated by armed forces	22	24.90	2.65	44
Non-EU Students	19	26.27	2.68	22

Note: Demographic data of *n* = 55 students was not available. These students were not included in the final analysis.

**Table 2:** Overall NTS performance of all students.

AS-NTS Score	ACLS I MV (SD)	ACLS II MV (SD)	OR-SIM MV (SD)	ACLS III MV (SD)
Sum_score of AS-NTS	2.18 (0.80)	2.16 (0.83)	1.84 (0.68)	2.01 (0.71)
Dimension one Planning tasks, prioritising and problem-solving	2.18 (0.91)	2.23 (0.93)	1.93 (0.75)	2.04 (0.85)
Dimension two Teamwork and leadership	2.19 (0.88)	2.16 (0.92)	1.79 (0.78)	2.03 (0.82)
Dimension three Team orientation	2.18 (0.91)	2.08 (0.91)	1.81 (0.79)	1.97 (0.79)

Note: Abbreviations. AS-NTS = Anaesthesiology students’ non-technical skills. ACLS = Advanced Cardiac Life Support. OR = Operating room.

**Table 3:** NTS performance of the admission cohorts for each training session and each dimension of the AS-NTS.

Admission	ACLS I			ACLS II			OR-SIM			ACLS III		
	D1	D2	D3	D1	D2	D3	D1	D2	D3	D1	D2	D3
**Excellent PEA**	2.28	2.28	2.28	2.07	2.14	2.02	1.80	1.70	1.60	2.10	2.01	1.95
**Ham-NAT**	2.19	2.17	2.19	2.30	2.21	2.21	2.03	1.92	1.86	2.05	2.01	1.98
**Ham-INT**	2.23	2.23	2.18	2.36	2.21	1.95	1.69	1.62	1.92	2.04	1.96	1.93
**Waiting quota**	1.86	1.95	2.00	1.75	1.73	1.75	1.73	1.69	1.65	1.86	1.93	1.79
**Other quota**	2.87	2.53	2.60	2.36	2.14	2.06	2.22	2.00	2.00	2.11	2.18	1.87
**Designated by the Armed Forces**	1.75	1.75	1.83	2.10	1.90	1.70	2.50	1.50	2.50	1.53	1.67	1.87
**Non-EU Students**	2.67	2.67	1.67	2.89	3.11	3.22	2.00	2.00	2.50	2.38	2.38	2.23

Note. Abbreviations. AS-NTS = Anaesthesiology students’ non-technical skills. ACLS = Advanced Cardiac Life Support. OR = Operating room. D = Dimension of AS-NTS. D1: Planning tasks, prioritising and problem-solving; D2: Teamwork and leadership; D3: Team orientation.

## Data Availability

Most data are presented in the manuscript. Further information can be provided due to reasonable request.
